# Supplementary material for: High-resolution monthly precipitation and temperature time series from 2006 to 2100
Source: Sci Data. 2020 Jul 23;7:248. doi: 10.1038/s41597-020-00587-y (PMC7378208; doi:10.1038/s41597-020-00587-y)
Supplement: Supplementary file 1 — Supplemental Information [file 41597_2020_587_MOESM1_ESM.pdf]

Supplementary Information

High resolution monthly precipitation and temperature timeseries for the period 2006-2100

Dirk Nikolaus Karger[1], Dirk Schmatz[1], Gabriel Dettling[1], Niklaus E. Zimmermann[1]

[1]. Swiss Federal Research Institute for Forest, Snow and Landscape WSL, Zürcherstrasse 111, 8903 Birmensdorf, Switzerland.

corresponding author(s): Dirk Nikolaus Karger (dirk.karger@wsl.ch)

Table of Contents

Figure S1. Mean absolute error (MAE), root mean square error (RSME), and spearman correlation (COR) of the downscaled mean monthly daily maximum temperatures (tmax) at 0.043° resolution (orange) and the original input from the four GCMs (blue) for a comparison with observations from GHCN.......... 2

Figure S2. Mean absolute error (MAE), root mean square error (RSME), and spearman correlation (COR) of the downscaled mean monthly daily minimum temperatures (tmin) at 0.043° resolution (orange) and the original input from the four GCMs (blue) for a comparison with observations from GHCN.......... 3

Figure S3. Mean absolute error (MAE), root mean square error (RSME), and spearman correlation (COR) of the downscaled precipitation (ps) at 0.043° resolution (orange) and the original input from the four GCMs (blue) for a comparison of mean monthly precipitation sums with observations from GHCN. 4

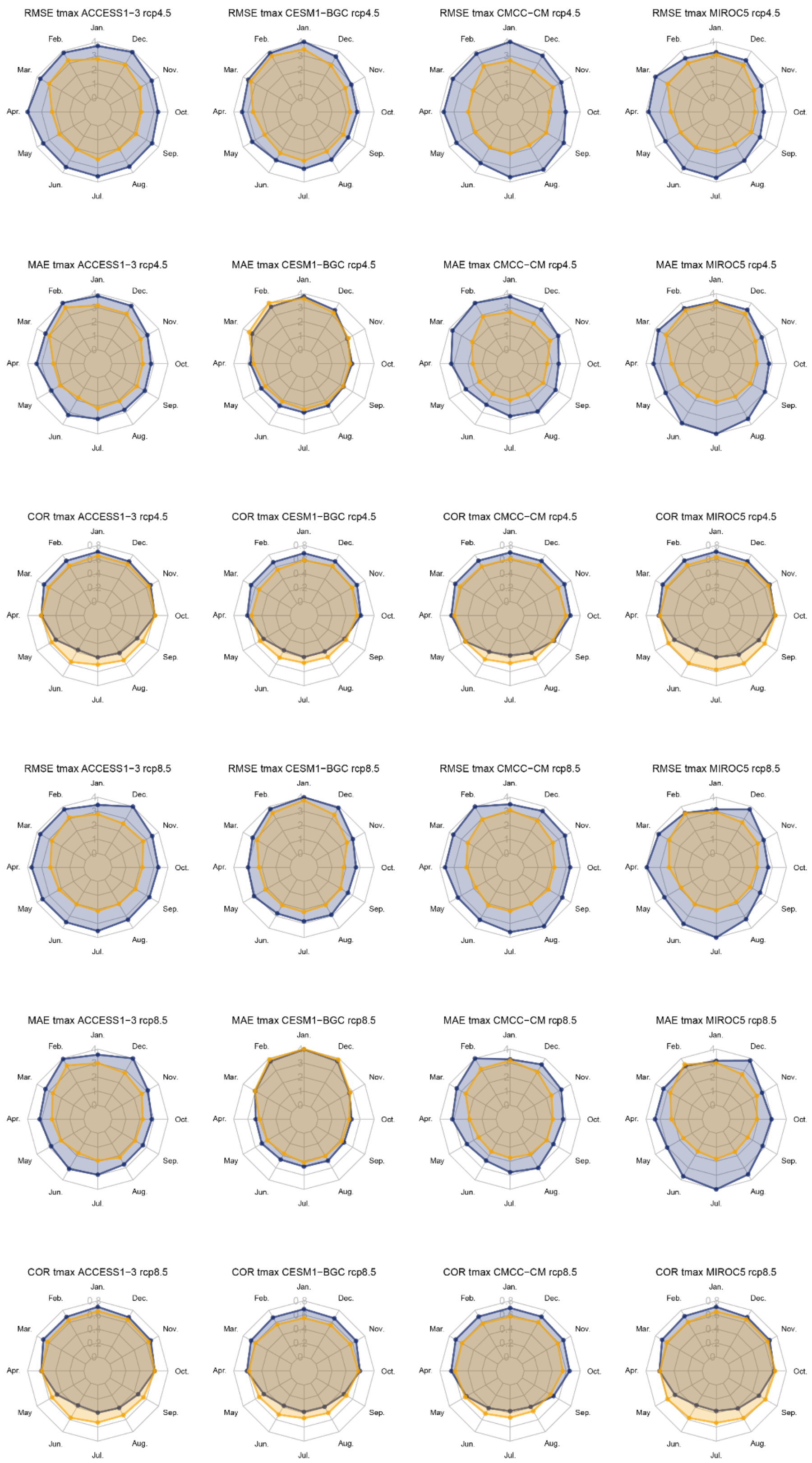

Figure S1. Mean absolute error (MAE), root mean square error (RSME), and spearman correlation (COR) of the downscaled mean monthly daily maximum temperatures (tmax) at 0.043° resolution (orange) and the original input from the four GCMs (blue) for a comparison with observations from GHCN.

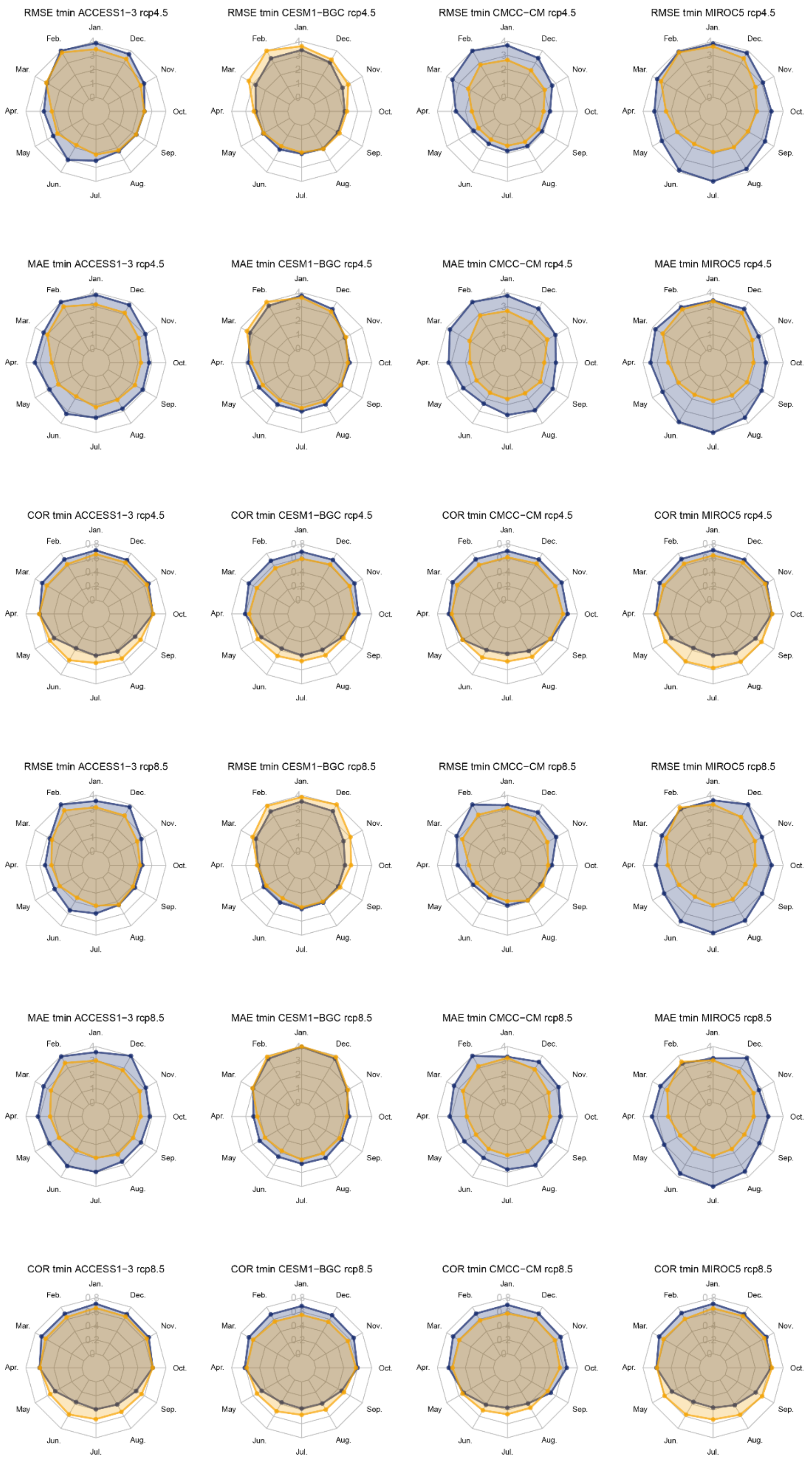

Figure S2. Mean absolute error (MAE), root mean square error (RSME), and spearman correlation (COR) of the downscaled mean monthly daily minimum temperatures (tmin) at 0.043° resolution (orange) and the original input from the four GCMs (blue) for a comparison with observations from GHCN.

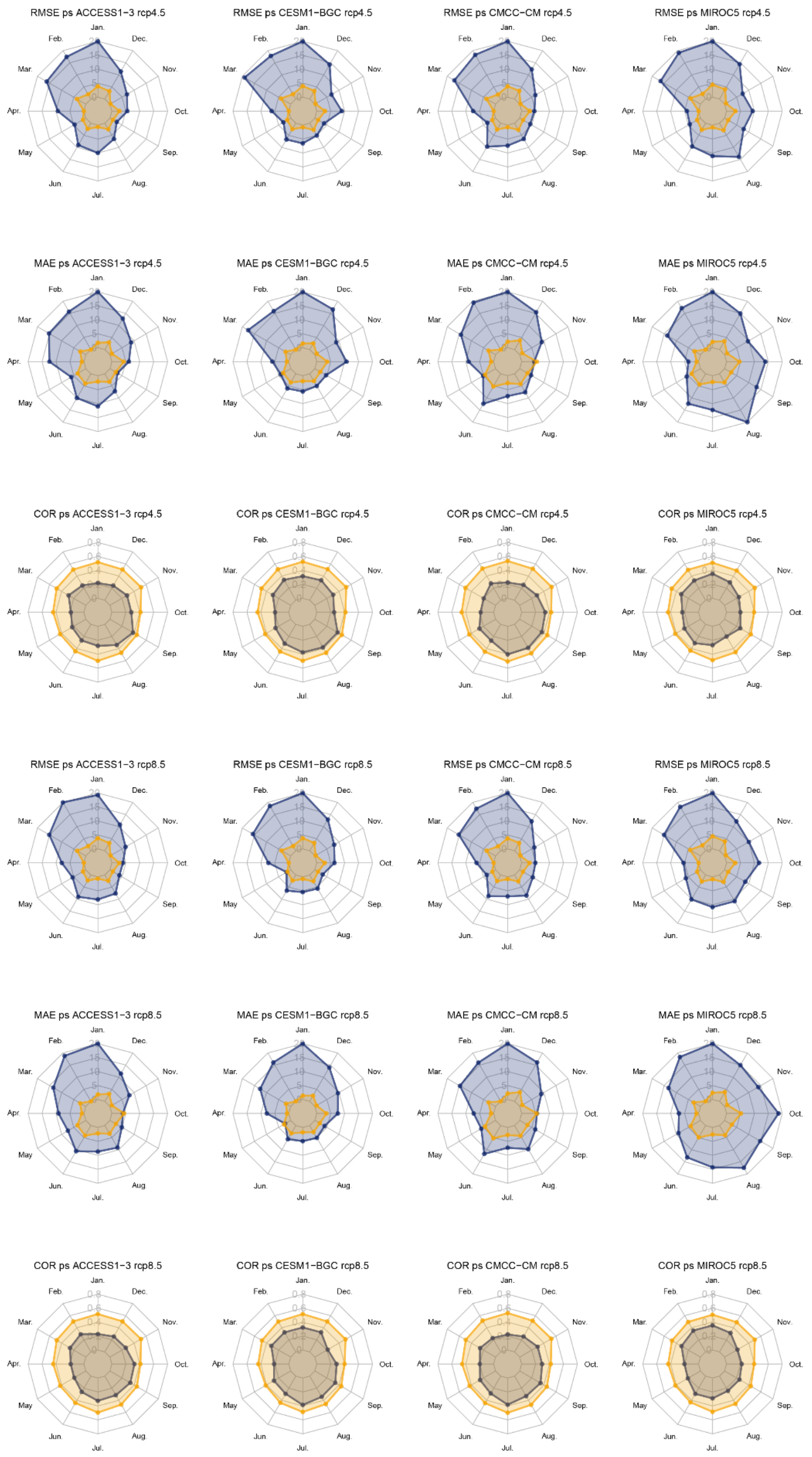

Figure S3. Mean absolute error (MAE), root mean square error (RSME), and spearman correlation (COR) of the downscaled precipitation (ps) at 0.043° resolution (orange) and the original input from the four GCMs (blue) for a comparison of mean monthly precipitation sums with observations from GHCN.
